# Cannabis-Associated Pneumothorax: A Case Report

**DOI:** 10.7759/cureus.50825

**Published:** 2023-12-20

**Authors:** Dennis N Mensah, Jonathan Livingston, Vasudha Maddukuri

**Affiliations:** 1 Internal Medicine, New York Medical College at St. Mary’s General Hospital and St. Clare’s Health, Denville, USA; 2 Pulmonary and Critical Care Medicine, Johns Hopkins University School of Medicine, Baltimore, USA

**Keywords:** tobacco vs cannabis, effects of cannabis use, pneumothorax, cannabis-associated pneumothorax, general internal medicine, cardiothoracic surgery, primary spontaneous pneumothorax, spontaneous pneumothorax, marijuana and pneumothorax

## Abstract

The use of cannabis for therapeutic and recreational purposes has been on the rise in recent years. This has increased the prevalence of cannabis use disorder across various demographic subgroups. A recent medical literature review describes a few cases demonstrating the association of spontaneous pneumothorax and bullous lung disease in cannabis users without concomitant tobacco use. We herein present a case report of a young male with chronic cannabis use who presented with right-sided spontaneous pneumothorax and bilateral apical blebs.

## Introduction

Primary spontaneous pneumothorax generally presents as an acute and debilitating medical condition with symptoms including dyspnea and pleuritic chest pain. Patients may present with cardiorespiratory collapse depending on the severity of the pneumothorax. Risk factors include tobacco smoking, asthenic body habitus, and young age. Males are more commonly affected than females, with an incidence ratio of 5.9 to 1, respectively [1-2]. Tobacco smoking has historically been linked to bullous lung disease, leading to spontaneous pneumothorax.

Additionally, reported studies have shown that concurrent tobacco and cannabinoid use increases the risk of spontaneous pneumothorax [3]. However, no conclusive evidence in studies to date has been established conferring cannabis use as an independent risk factor for spontaneous pneumothorax. With the growing trends in cannabis use and the recent legalization across several states in America, we predict an increase in the number of cannabinoid-associated pneumothorax; however, further research is warranted to substantiate the potential detriment of chronic cannabis use. In this case, we present a 23-year-old male who developed a spontaneous pneumothorax in the setting of chronic cannabis use without a history of tobacco consumption.

## Case presentation

**Figure 1:**
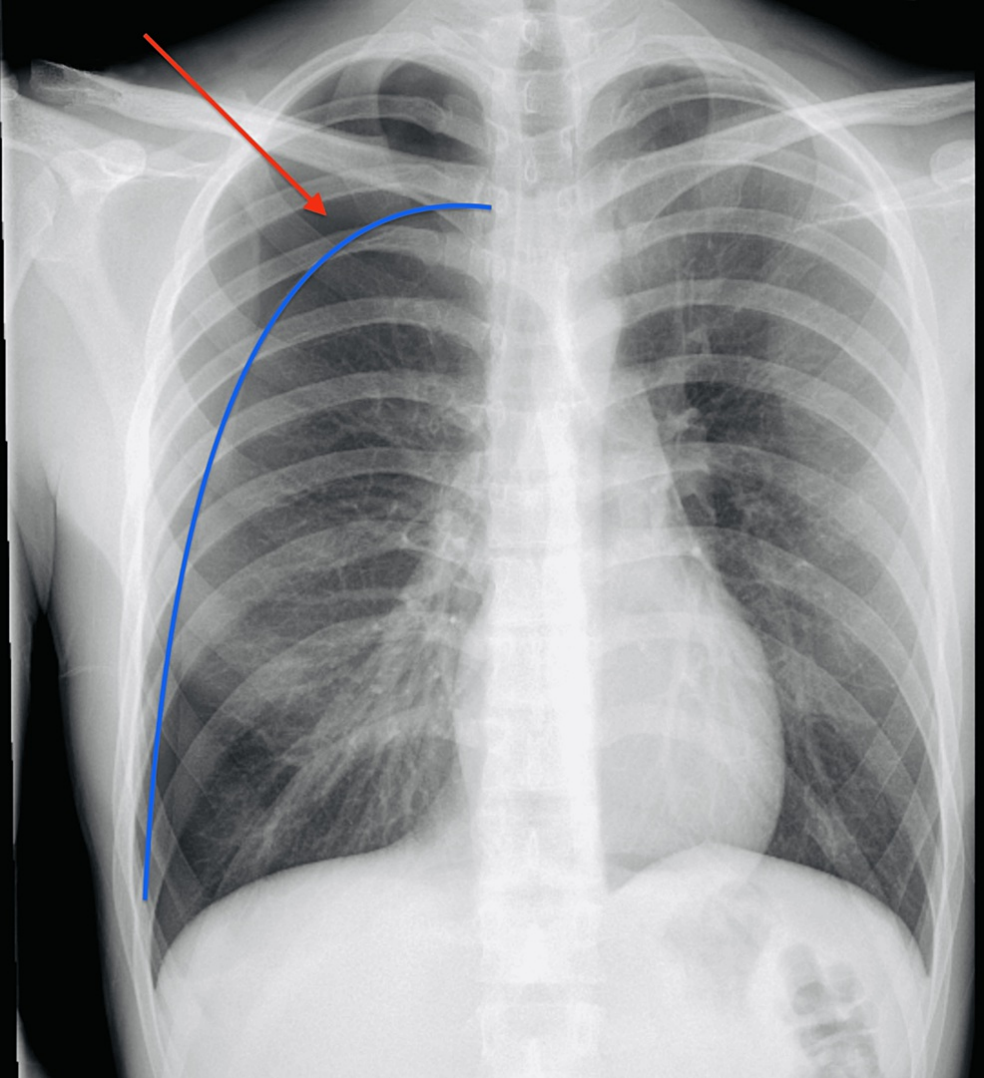
Chest X-ray Initial Chest X-ray film revealed a moderate right-sided spontaneous pneumothorax (red arrow) without evidence of tension or mediastinal shift. (Blue line) delineating the junction between the pneumothorax and lung parenchyma.

A 23-year-old male with a past medical history of asthma presented to the emergency department complaining of acute right-sided chest pain. The onset of pain was sudden and described as sharp and pleuritic in nature. The intensity of the pain was rated as a 10 on a pain scale of 10 and was exacerbated by deep breathing. He denied trauma, travel, recent or recurrent infections, or exposure to sick contacts. His last asthma exacerbation was at age seven, and he has not required prescription medications since that time. The patient denied tobacco use but endorsed a several-year history (greater than five years) of smoking cannabis in the form of joints, approximately 1-3 joints per day. He denied using vapes or other pipe-smoking paraphernalia. The patient had no prior history of similar symptoms. He denied having any predisposing familial genetic diseases. On physical examination, the patient was afebrile and normotensive with a blood pressure of 114/63 mmHg, heart rate of 63 beats per minute, and respiratory rate of 20 breaths per minute. His oxygen saturation was 100% on ambient air. The pertinent diagnostic workup included a chest x-ray, which revealed findings consistent with a moderate right-sided spontaneous pneumothorax without evidence of tension or mediastinal shift, as shown in (Figure 1).

**Figure 2:**
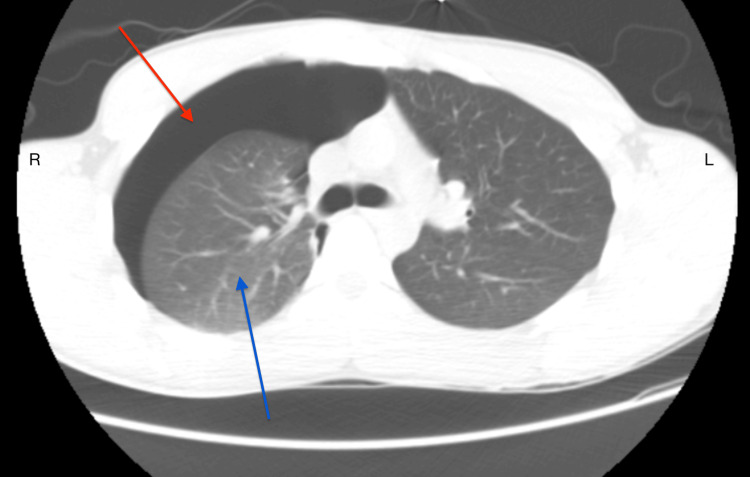
Non-contrast CT image prior to chest tube placement Before chest tube placement, a pre-procedural axial plane image revealed a right-sided pneumothorax (red arrow) and normal lung parenchyma (blue arrow).

Initial management consisted of a non-contrast computed tomography (CT) guided tube thoracostomy. Pre-procedural imaging revealed a moderate-to-large right-sided pneumothorax (Figure 2).

**Figure 3:**
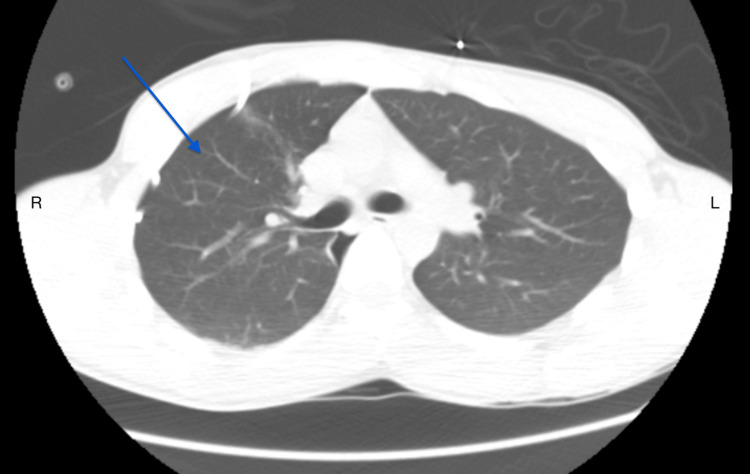
Non-Contrast CT Image After Chest Tube Placement Post-procedural axial plane image after chest tube placement showing approximately 95% lung re-expansion of the right-sided pneumothorax (blue arrow).

Post-procedural imaging showed approximately 95% lung re-expansion with the chest tube catheter left in place to suction via the Pleur-evac container shown in (Figure 3).

During the hospitalization, the patient was found to have a persistent air leak suspected to be caused by a ruptured bleb at the right lung apex. The patient was seen and evaluated by cardiothoracic surgery and ultimately underwent video-assisted thoracoscopic resection of right upper lobe blebs. The operative report described the visual inspection of the lung field to show areas of exudate and fibrin, presumably from a ruptured bulla with exudate and evidence of secondary inflammation. The patient had an uncomplicated postoperative course with serial chest X-rays showing no further persistence of a pneumothorax. He was ultimately discharged in stable condition on postoperative day five.

## Discussion

Cannabis is one of the most commonly used psychoactive substances in the United States, with over 48 million people age 12 or older reporting past-year cannabis use in 2019. This is mainly attributed to the medicinal and recreational legalization of cannabis in many states. To date, medical use of cannabis is legal in 40 states and the District of Columbia, whereas recreational or adult use is approved in 23 states and the District of Columbia [4]. Although the association between tobacco use and bullous lung disease leading to pneumothorax is well established, the potential for cannabis use as an independent risk factor has not yet been substantiated. Nevertheless, there have been some cases reported in recent literature describing an increasing trend in spontaneous pneumothorax from bullous disease in patients with reported combined cannabis and tobacco use [5]. 

Various pathophysiologic mechanisms have been postulated regarding cannabinoid-associated pneumothorax. The deleterious effect of carcinogenic compounds in cannabis on the lungs, deep inhalation, and longer breath-holding during cannabis use makes patients prone to developing barotrauma, leading to spontaneous pneumothorax resulting from high inspiratory pressures [4,6]. Cannabis can also affect lung function and mechanics by increasing forced vital capacity, total lung capacity, functional residual capacity, and residual volume. Together, these changes may increase the predisposition of the lungs to air trapping and subsequent lung hyperinflation and alveolar rupture [7]. Hii et al. demonstrated this effect of cannabis use on the lungs in a case series of 10 patients who were chronic cannabis smokers and developed bullous lung disease on a high-resolution CT scan [8].

The case presented in this report highlights a young patient with chronic cannabis use without a tobacco history who developed a spontaneous right-sided pneumothorax. Chest computed tomography (CT) showed right apical blebs for which he underwent video-assisted thoracoscopy (VATS) and bleb resections. The pattern of clinical presentation in our patient is comparable to a prior case published that reported an 18-year-old male who developed a spontaneous pneumothorax after a long-standing history of heavy cannabis use. He underwent VATS after CT imaging showed bilateral bullae at the lung apices [9]. In a case series by Johnson et al., chronic cannabis use was presumed to be the common variable amongst the patients who developed bullous lung disease with subsequent spontaneous pneumothorax [10]. There was a history of co-existing tobacco use among the patients in Johnson’s case series, albeit to a lesser degree in contrast to our patient. In an additional case report by Mishra et al., a 30-year-old patient who solely smoked cannabis daily for five years was found to have a spontaneous pneumothorax in the setting of right apical lung bullae [11].

Another potential cause of spontaneous pneumothorax is genetic factors consisting of inherited familial or sporadic mutations. The tumor suppressor gene FLCN, which encodes for the folliculin protein, is implicated in both genetic mutation manifestations. In addition, spontaneous pneumothorax has been associated with several genetic syndromes, including but not limited to Birt-Hogg-Dubé syndrome, tuberous sclerosis, Ehlers-Danlos syndrome, and Marfan syndrome [12]. Our patient denied a family history of pneumothorax and did not exhibit any additional genetic syndromic characteristics on physical examination; thus, genetic testing was deferred. However, outpatient testing may be recommended for a complete comprehensive workup, which may help elucidate the potential for a recurrent spontaneous pneumothorax in the future. 

Cannabis use may have a synergistic effect with tobacco use or may be confounded by the influence of tobacco use in its association with pneumothorax. Nonetheless, cannabis smoke may act independently to cause lung toxicities and alter lung function and pathophysiological changes, leading to bullous lung disease and, subsequently, a pneumothorax [3,13]. This underscores the importance of further epidemiology investigations, which are limited in the literature at this time about the incidence of spontaneous pneumothorax in the setting of independent cannabis use.

## Conclusions

A persistent rise in the incidence of bullous lung disease and spontaneous pneumothorax has been observed among individuals exhibiting chronic cannabis consumption patterns. To substantiate these purported associations, further systematic investigation is imperative. Healthcare practitioners are encouraged to exercise enhanced diligence in recognizing such associations and incorporate inquiries concerning cannabis utilization within the medical history assessment of patients presenting with spontaneous pneumothorax. The following case report underscores the potential peril of cannabis use as an independent risk factor for bullous lung disease, which may culminate in the development of a spontaneous pneumothorax, thereby posing a life-threatening concern.

## References

[1] (2023). Substance Abuse and Mental Health Services Administration: 2019 NSDUH Detailed Tables. https://www.samhsa.gov/data/report/2019-nsduh-detailed-tables.

[2] Olesen WH, Titlestad IL, Andersen PE, Lindahl-Jacobsen R, Licht PB (2019). Incidence of primary spontaneous pneumothorax: a validated, register-based nationwide study. ERJ Open Res.

[3] Hedevang Olesen W, Katballe N, Sindby JE (2017). Cannabis increased the risk of primary spontaneous pneumothorax in tobacco smokers: a case-control study. Eur J Cardiothorac Surg.

[4] Hasin D, Walsh C (2021). Trends over time in adult cannabis use: a review of recent findings. Curr Opin Psychol.

[5] Bisconti M, Marulli G, Pacifici R (2019). Cannabinoids identification in lung tissues of young cannabis smokers operated for primary spontaneous pneumothorax and correlation with pathologic findings. Respiration.

[6] Wu TC, Tashkin DP, Djahed B, Rose JE (1988). Pulmonary hazards of smoking marijuana as compared with tobacco. N Engl J Med.

[7] Hancox RJ, Poulton R, Ely M (2010). Effects of cannabis on lung function: a population-based cohort study. Eur Respir J.

[8] Hii SW, Tam JD, Thompson BR, Naughton MT (2008). Bullous lung disease due to marijuana. Respirology.

[9] Allen RK (2010). Bullectomy for ‘bong lung’ in an 18 year-old male presenting with spontaneous pneumothorax. Pneumon.

[10] Johnson MK, Smith RP, Morrison D, Laszlo G, White RJ (2000). Large lung bullae in marijuana smokers. Thorax.

[11] Mishra R, Patel R, Khaja M (2017). Cannabis-induced bullous lung disease leading to pneumothorax: case report and literature review. Medicine (Baltimore).

[12] Boone PM, Scott RM, Marciniak SJ, Henske EP, Raby BA (2019). The Genetics of pneumothorax. Am J Respir Crit Care Med.

[13] Feldman AL, Sullivan JT, Passero MA (1993). Pneumothorax in polysubstance-abusing marijuana and tobacco smokers: three cases. J Subst Abuse.

